# Variants in the *AGBL5* gene are responsible for autosomal recessive Retinitis pigmentosa with hearing loss

**DOI:** 10.1038/s41431-024-01768-8

**Published:** 2024-12-13

**Authors:** Marianthi Karali, Gema García-García, Karolina Kaminska, Alaa AlTalbishi, Francesca Cancellieri, Francesco Testa, Maria Rosaria Barillari, Evangelia S. Panagiotou, George Psillas, Veronika Vaclavik, Viet H. Tran, Lucas Janeschitz-Kriegl, Hendrik PN Scholl, Manar Salameh, Pilar Barberán-Martínez, Ana Rodríguez-Muñoz, Miguel Armengot, Margherita Scarpato, Roberta Zeuli, Mathieu Quinodoz, Francesca Simonelli, Carlo Rivolta, Sandro Banfi, José M. Millán

**Affiliations:** 1 Medical Genetics, Department of Precision Medicine, University of Campania ‘Luigi Vanvitelli’, 80138 Naples, Italy; 2 Eye Clinic, Multidisciplinary Department of Medical, Surgical and Dental Sciences, University of Campania ‘Luigi Vanvitelli’, 80131 Naples, Italy; 3 Molecular, Cellular, and Genomic Biomedicine Group, IIS-La Fe, Valencia, Spain; 4 Center for Rare Diseases (CIBERER), Madrid, Spain; 5 Joint Unit CIPF-IIS La Fe Molecular, Cellular, and Genomic Biomedicine, Valencia, Spain; 6 Institute of Molecular and Clinical Ophthalmology Basel, 4031 Basel, Switzerland; 7 St John of Jerusalem Eye Hospital, Jerusalem, Palestine; 8 Department of Mental and Physical Health and Preventive Medicine, University of Campania ‘Luigi Vanvitelli’, 80138 Naples, Italy; 9 1st Department of Ophthalmology, Aristotle University of Thessaloniki, AHEPA Hospital, Thessaloniki, Greece; 10 Department of Ophthalmology, Ghent University Hospital, Ghent, Belgium; 11 1st Academic ENT Department, School of Medicine, Aristotle University of Thessaloniki, AHEPA Hospital, Thessaloniki, Greece; 12 Jules-Gonin Eye Hospital, Fondation Asile des Aveugles, University of Lausanne, 1004 Lausanne, Switzerland; 13 Centre for Gene Therapy and Regenerative Medicine, King’s College London, London, UK; 14 Department of Ophthalmology, University of Basel, Basel, Switzerland; 15 University Dr Peset Hospital of Valencia, Valencia, Spain; 16 University and Polytechnic La Fe Hospital of Valencia, Valencia, Spain; 17 Department of Genetics and Genome Biology, University of Leicester, Leicester, UK; 18 Telethon Institute of Genetics and Medicine, 80078 Pozzuoli, Italy

**Keywords:** Genetics research, Disease genetics, Medical genetics

## Abstract

The *AGBL5* gene encodes for the Cytoplasmic Carboxypeptidase 5 (CCP5), an α-tubulin deglutamylase that cleaves the γ-carboxyl-linked branching point of glutamylated tubulin. To date, pathogenic variants in *AGBL5* have been associated only with isolated retinitis pigmentosa (RP). Hearing loss has not been reported in *AGBL5*-caused retinal disease. In this study, we performed exome sequencing in probands of eight unrelated families from Italy, Spain, Palestine, Switzerland, and Greece. All subjects had a clinical diagnosis of (suspected) Usher syndrome type II for the concurrent presence of RP and post-verbal sensorineural hearing loss (SNHL) that ranged from mild to moderate.We identified biallelic sequence variants in *AGBL5* in all analysed subjects. Four of the identified variants were novel. The variants co-segregated with the retinal and auditory phenotypes in additional affected family members. We did not detect any causative variants in known deafness or Usher syndrome genes that could explain the patients’ hearing loss. We therefore conclude that SNHL is a feature of a syndromic presentation of *AGBL5* retinopathy. This study provides the first evidence that mutations in *AGBL5* can cause syndromic RP forms associated with hearing loss, probably due to dysfunction of sensory cilia in the retina and the inner ear.

## Introduction

Retinitis pigmentosa (RP) is the most common form of inherited retinal dystrophy (IRD) with an incidence of 1:3500–1:4000 [1]. The main cellular hallmark of RP is a progressive degeneration of photoreceptors that leads first to night-blindness and loss of peripheral visual field, and later to blindness [1]. RP is more frequently observed as an isolated form (also called simplex RP) [2], however, in a third of cases it occurs in the context of syndromic conditions with extraretinal involvement [2]. Examples of syndromic RP conditions include Usher, Alstrom, Bardet-Biedl syndromes as well as mitochondrial and peroxisomal disorders. These syndromes are characterized by retinal dystrophy associated with additional features such as hearing loss, kidney defects, polydactyly, and obesity among others [3]. Overall, hearing loss is the most frequent extraocular feature in syndromic forms, along with central nervous system defects [4]. To date, 281 genes have been associated with IRDs (RetNet, http://web.sph.uth.edu/RetNet/; January 2024). Pathogenic variants in the *AGBL5* gene (OMIM * 615900), also known as *CCP5*, have been reported to cause isolated autosomal recessive retinitis pigmentosa (arRP) in different populations [5–14]. To the best of our knowledge, only 22 patients from 16 unrelated families with biallelic variants in *AGBL5* have been reported thus far.

*AGBL5* encodes for the protein Cytoplasmic Carboxypeptidase 5 (CCP5), an α-tubulin deglutamylase [15]. Tubulin deglutamylases (CCP1-6) are members of the M14 CCP protein family, alternatively known as ATP/GTP-binding protein-like family [16]. CCP5 is a cilia-enriched deglutamylase and has a specialized role among CCPs: while most CCPs shorten the long glutamate chains, CCP5 is the only deglutamylase that can specifically cleave the γ-carboxyl-linked branching point of the glutamylated tubulin [15, 17]. The glutamylated C-terminal end of tubulins creates binding sites for microtubule interacting proteins. Consequently, proper tubulin glutamylation is critical for the maintenance and function of several organelles that rely on microtubule-based structures (such as cilia, neurons, and centrioles) and its perturbation induces a range of ciliopathy phenotypes [18]. Specifically, hyperglutamylation due to *ccp5* knockdown in morphant zebrafish causes axis curvature, hydrocephalus, pronephric cysts, eye size reduction, and cilia motility defects [19, 20]. Ccp5 depletion in knock-out mice has no effect on primary cilia but compromises immune responses to viral infections and induces infertility due to defects in spermatogenesis [21–23]. Interestingly, depletion of *CCP5* in cells deficient for *ARL13B*, a gene associated with Joubert syndrome, restores proper levels of axoneme glutamylation and rescues defects in ciliary length and signaling [24].

Given the importance of AGBL5/CCP5 in maintaining proper tubulin glutamylation in ciliated cells, and considering the key role of the latter in sensory perception, it is tempting to speculate that mutations in *AGBL5* could lead to ciliopathy phenotypes with syndromic extraretinal manifestations. Here, we describe the clinical features of patients from eight unrelated families who presented syndromic RP with hearing loss and harbored biallelic pathogenic variants in *AGBL5*. Taken together, our results suggest that *AGBL5* is a new gene associated with an Usher-like phenotype. This work expands the phenotypic spectrum associated with mutations in *AGBL5* and describes in detail the ophthalmological and audiological features of affected subjects.

## Methods

### Subjects

Subjects reported in this study were diagnosed at the Referral Center for Inherited Retinopathies of the University of Campania ‘Luigi Vanvitelli’ (patients P1-IT, P2-IT), the Hospital La Fe (P3-ES), the St John of Jerusalem Eye Hospital (P6-PS, P7-PS), the Hôpital ophtalmique Jules Gonin in Lausanne (P8-CH), the Basel University Hospital Eye clinic (P9-CH), and the 1st Department of Ophthalmology of the Aristotle University of Thessaloniki (P10-GR). All procedures adhered to the tenets of the Declaration of Helsinki and were approved by the Ethics Committees of the participating institutes. An informed consent and a medical history questionnaire were completed by all patients. Peripheral blood samples or saliva specimens were collected upon written informed consent of the subjects to sample collection and genetic analysis.

### Ophthalmological examination

Subjects underwent a complete ophthalmological assessment which included best-corrected visual acuity (BCVA) measurements, slit lamp examination of anterior segment, intraocular pressure, fundus examination, Goldmann visual field (GVF), spectral domain optical coherence tomography (SD-OCT), fundus autofluorescence imaging (FAF) and standard electroretinography (ERG). GVF was measured by moving the III4e and V4e stimulus target on a calibrated standard Goldmann perimeter by experienced ophthalmic technicians. SD-OCT and FAF were performed with a Heidelberg Eye Explorer Version 1.9.11.0 (Heidelberg, Germany), an Optovue Solix (Pont-de-l’Arche, France), a Topcon Triton plus 3D (Tokyo, Japan) and a Nidek Mirante device (Gamagori, Japan) by experienced operators. ERG was performed following pupil dilation using 1% tropicamide and 2.5% phenylephrine hydrochloride, according to International Society for Clinical Electrophysiology of Vision (ISCEV) standards using an Espion Visual Electrophysiology System (Diagnosys Vision Ltd, Dublin, Ireland). The protocol included rod-specific and standard bright flash ERG, both recorded after a minimum of 20 min dark adaptation. Photopic 30 Hz flicker cone and transient cone ERGs were recorded following 10 min of light adaptation [25].

### Audiological examination

Audiological assessment included: evaluation of medical history, otoscopy (to exclude external and/or middle ear disease), pure-tone audiometry with evaluation of the Pure Tone Average (PTA), auditory brainstem response and other relevant audiological information. Pure tone audiometry was performed assessing the frequencies 125, 250, 500, 1000, 2000, 4000, 6000 and 8000 Hz for air conduction and 250, 500, 1000, 2000, 4000 Hz for bone conduction. PTA was calculated considering the thresholds at 250, 500, 1000, 2000, 4000 Hz. The degree of hearing loss severity according to PTA was defined according to the World Health Organization classification system i.e. *mild* (PTA 25–40 dB), *moderate* (PTA 41–60 dB), *severe* (PTA 61–80 dB) and *profound* (> 80 dB).

### Next Generation Sequencing and variant interpretation

Clinical exome (for P1-IT) and whole-exome sequencing (WES) protocols (for P2-IT, P3-ES, P6-PS, P7-PS, P8-CH, P9-CH, P10-GR) along with variant prioritisation strategies are detailed in Supplementary Methods. Alignments at candidate positions were visually inspected using the Integrative Genomics Viewer (IGV). Potentially pathogenic variants were confirmed by Sanger sequencing using the Big Dye Terminator (Applied Biosystems, Waltham, MA, USA) kit on an ABI3130xl (Applied Biosystems, Waltham, MA, USA) capillary sequencer or performed by Microsynth (Balgach, Switzerland).

## Results

### Clinical and genetic findings

In the course of the diagnostic workup to identify the genetic defects in IRD subjects, we analysed the probands of eight unrelated families of Italian, Spanish, Palestinian, Swiss, and Greek origin. All subjects presented with hearing loss in association with typical RP and were diagnosed with suspected Usher syndrome. We performed targeted NGS analysis using a clinical exome panel and/or WES, as detailed in Supplementary Methods, and identified biallelic variants in the *AGBL5* gene. These variants segregated with the disease phenotype in the index cases and additional members of their families according to a recessive pattern of inheritance. To detect causal variants that could account for the hearing deficit in these IRD patients, we interrogated an in silico panel of Usher syndrome and deafness-associated genes which, however, did not reveal any candidate pathogenic variants. Below we provide a detailed ophthalmological and audiological phenotyping of these patients. Genetic and clinical information for each patient are summarized in Table 1 and Table 2, respectively.

**Table 1:** Genetic findings in the *AGBL5* cohort.

Region of Origin	Family ID	Patient ID	Sex	Age	Genotype	Allele 1			Allele 2		
						Nucleotide^#^	Protein	ACMG class^$^	Nucleotide^#^	Protein	ACMG class^$^
Italy	F1	P1 - IT	Female	55	Hom	c.323C>G	p.(Pro108Arg)	Likely Pathogenic	c.323C>G	p.(Pro108Arg)	Likely Pathogenic
Italy	F2	P2 - IT	Male	42	Hom	c.184_185del	p.(Ala63Ter)	Likely Pathogenic	c.184_185del	p.(Ala63Ter)	Likely Pathogenic
Spain	F3	P3 - ES	Female	65	Comp. Het	c.908G>A	p.(Arg303His)	Likely Pathogenic	c.1565del	p.(Gly522AspfsTer4)	Likely Pathogenic
Spain	F3	P4 - ES	Male	69	Comp. Het	c.908G>A	p.(Arg303His)	Likely Pathogenic	c.1565del	p.(Gly522AspfsTer4)	Likely Pathogenic
Spain	F3	P5 - ES	Male	51	Comp. Het	c.908G>A	p.(Arg303His)	Likely Pathogenic	c.1565del	p.(Gly522AspfsTer4)	Likely Pathogenic
Palestine	F4	P6 - PS	Male	27	Hom	c.170G>A	p.(Trp57Ter)	Likely Pathogenic	c.170G>A	p.(Trp57Ter)	Likely Pathogenic
Palestine	F5	P7 - PS	Male	28	Hom	c.170G>A	p.(Trp57Ter)	Likely Pathogenic	c.170G>A	p.(Trp57Ter)	Likely Pathogenic
Switzerland	F6	P8 - CH	Female	36	Hom	c.908G>A	p.(Arg303His)	Likely Pathogenic	c.908G>A	p.(Arg303His)	Likely Pathogenic
Switzerland	F7	P9 - CH	Male	39	Hom	c.908G>A	p.(Arg303His)	Likely Pathogenic	c.908G>A	p.(Arg303His)	Likely Pathogenic
Greece	F8	P10 - GR	Female	63	Comp. Het	c.883G>A	p.(Asp295Asn)	Likely Pathogenic	c.316A>G	p.(Met106Val)	VUS

^#^nucleotide position is reported for the NM_021831.6 RefSeq transcript.

^$^according to the automated annotation in Varsome.

**Table 2:** Ophthalmological and audiological findings in the *AGBL5* index cases.

				Ophthalmological findings							Audiological findings				
	Family ID	Patient ID	Clinical diagnosis	Symptoms onset	Visual Field	Visual acuity	Fundus	Macular involvement	Lens	ERG	HL onset	HL type and severity	PTA	Frequencies	Progression
	F1	P1 - IT	RP and hearing loss	14 y	n.a.	HM (RE, LE)	vessel attenuation, diffuse RPE dystrophy, BSP in mid-periphery	atrophy	pseudophakia	unrecordable	18 y	moderate SNHL	59 dB (RE, LE)	bilateral, symmetric; sloping audiogram; mainly intermediate-high frequencies (500–8000 Hz)	no
	F2	P2 - IT	RP and hearing loss	14 y	5° central (RE), n.a. (LE)	0.2 D (RE), HM (LE)	waxy ONH palor, vessel attenuation, diffuse RPE dystrophy, BSP in mid-periphery	atrophy	pseudophakia	unrecordable	post-verbal	mild-to-moderate SNHL	39 dB (RE), 38 dB (LE)	bilateral, symmetric; intermediate frequencies (500–2000 Hz)	no
	F3	P3 - ES	RP and hearing loss	>10 y	<10° (RE, LE)	0.3 D (RE, LE)	waxy ONH palor, vessel attenuation, pigment deposits in periphery	dystrophy	pseudophakia	n.a.	50 y	moderate SNHL	50 dB (RE), 49 dB (LE)	bilateral, symmetric; sloping audiogram; mainly intermediate-high frequencies (500–8000 Hz)	no
	F4	P6 - PS	RP and hearing loss	n.a.	n.a.	0.5 D (RE, LE)	vessel attenuation, no BSP	CME	posterior subcapsular cataract	unrecordable	7 y	mild-to-moderate SNHL	39 dB (RE), 46 dB (LE)	low-to-mid frequencies	n.a.
	F5	P7 - PS	RP and hearing loss	n.a.	n.a.	0.16 D (RE, LE)	vessel attenuation, BSP	no	posterior subcapsular cataract	unrecordable	5 y	moderate SNHL	49 dB (RE, LE)	mild in low and high frequencies, moderate in mid frequencies	n.a.
	F6	P8 - CH	RP and hearing loss	14 y	<15° (RE, LE)	0.1 D (RE, LE)	foveal and peripapillary atrophy, pathes of atrophy in mid-periphery	atrophy	pseudophakia	unrecordable	not perceived by the patient	subclinical SNHL	19 dB (RE), 15 dB (LE)	high frequencies	n.a.
	F7	P9 - CH	RP and hearing loss	20 y	<10° visual field (LE); RE enucleated due to phtisis following retinal detachments in early adulthood	1.5 D (LE)	waxy ONH palor, vessel attenuation, peripheral BSP and retinal atrophy	no	pseudophakia	n.a.	congenital	moderate-to-severe SNHL	65 dB (RE), 75 dB (LE)	moderate in low-to-mid frequencies, severe in high frequencies	no
	F8	P10 - GR	RP and hearing loss	25 y	n.a.	0.16 D (RE), 0.125 D (LE)	vessel attenuation, peripheral outer retinal atrophy and BSP	atrophy	pseudophakia	n.a.	58 y	moderate SNHL	51 dB (RE), 50 dB (LE)	bilateral, flat audiogram	no

*BSP* Bone spicule pigmentation, *CME* Cystoid macular edema, *D* Decimal, *HL* hearing loss, *HM* Hand motion, *LE* Left eye/ear, *n.a.* not available, *ONH* optic nerve head, *PTA* Pure tone average, *RE* Right eye/ear, *RPE* Retinal pigment epithelium, *SNHL* sensorineural hearing loss.

### Index case P1-IT (family F1)

**Fig. 1:**
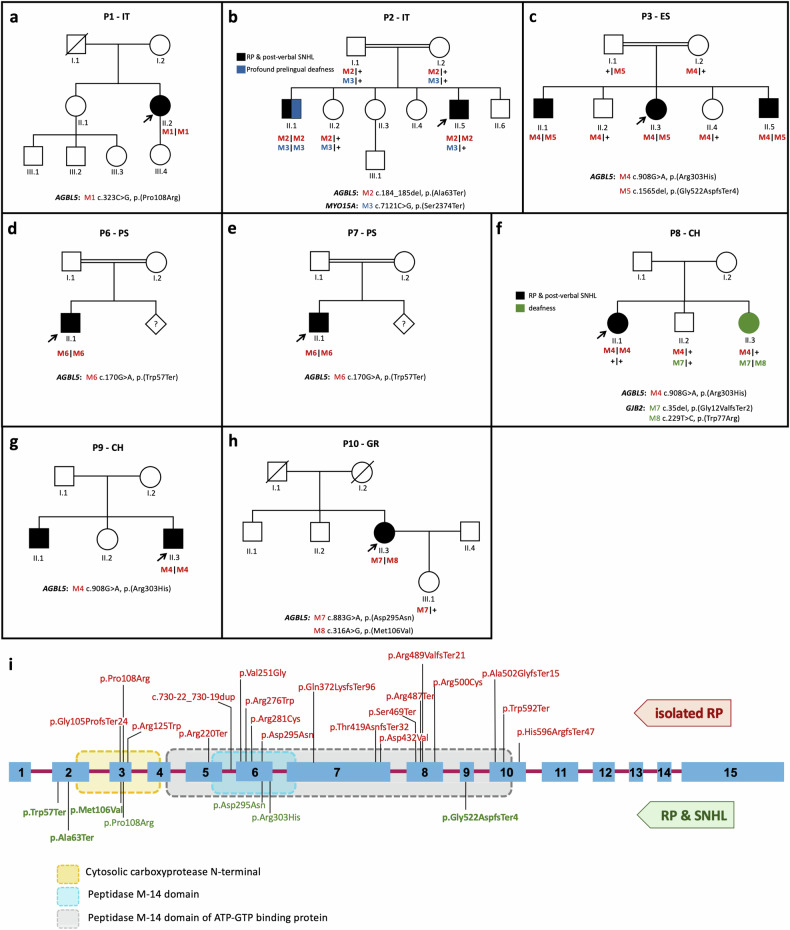
Pedigree structure and variants identified in the *AGBL5* cohort. **a–h** Pedigrees of the families included in this study and segregation analysis of the identified disease-causing variants. Probands are indicated by arrows. Filled symbols denote affected individuals and color indicates phenotype (black, RP and post-verbal SNHL; blue, profound prelingual deafness; green, isolated deafness). Variant positions refer to the RefSeq transcript sequences NM_021831.6 (*AGBL5*), NM_016239.4 (*MYO15A*) and NM_004004.6 (*GJB2*). **i** Schematic representation of the relative position of the variants identified in this study (in green) across the *AGBL5* gene structure and in the context of the AGBL5 protein domains (dotted boxes). Novel variants are shown in bold. Variants already reported in the literature in cases with non-syndromic RP are shown in red font.

**Fig. 2:**
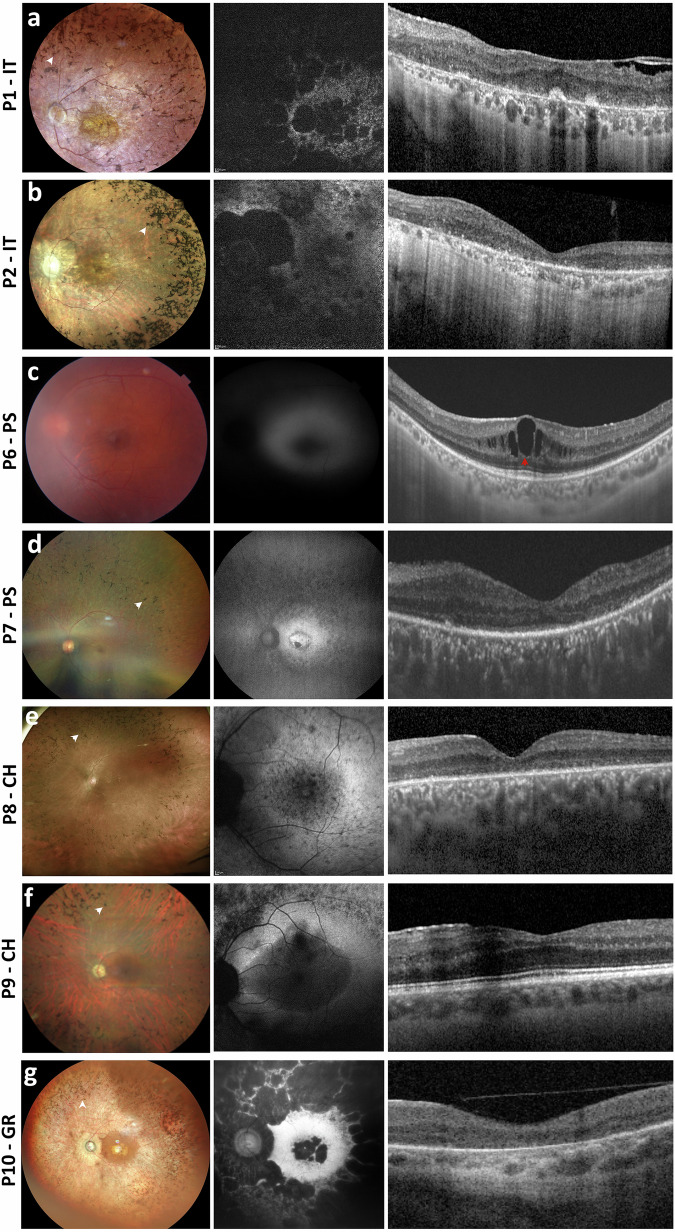
Multimodal ophthalmological imaging findings in the probands of this cohort. Fundus photograph (left side of each panel), fundus autofluorescence imaging (FAF, central image of each panel), and macular spectral domain optical coherence tomography (SD-OCT, right side of each panel) of the left eye of the probands included in this study. Bone-spicule pigment (BSP) in fundus photographs and cystoid macular edema (CME) in SD-OCT scans are indicated with white and red arrowheads, respectively. Stretches of retinal pigment epithelium (RPE) dystrophy or atrophy in SD-OCT scans are represented by bright areas of increased light transmission beneath the RPE. **a** Patient P1-IT: Fundus photograph showing RP fundus with diffuse dystrophy of the RPE, BSP in mid-periphery and macular atrophy. FAF imaging shows reduced autofluorescence at the posterior pole, with focal areas of absent autofluorescence surrounding the optic disc and at the macular area. SD-OCT scan shows RPE dystrophy with epiretinal membranes. **b** Patient P2-IT: Fundus photograph showing waxy optic nerve head (ONH) pallor, RP fundus with diffuse RPE dystrophy and BSP in mid-periphery. FAF imaging shows reduced autofluorescence at the posterior pole, with focal areas of absent autofluorescence surrounding the optic disc. OCT scan shows reduced retinal thickness and RPE atrophy at the macular area. **c** Patient P6-PS: Fundus photograph showing RPE dystrophy without BSP. FAF image suggesting reduced autofluorescence which could not be fully assessed due to lens opacities. OCT scan shows CME. **d** Patient P7-PS: Fundus photograph showing RPE dystrophy with BSP. FAF imaging showed focal areas of reduced autofluorescence in mid-periphery and in the macular area. OCT scan shows stretches of RPE dystrophy and atrophy. **e** Patient P8-CH: Fundus photograph showing RPE dystrophy and BSP. FAF image showing focal areas of reduced AF in mid-periphery and at the macular area. OCT shows RPE dystrophy. **f** Patient P9-CH: Fundus photograph showing waxy ONH pallor, typical RP fundus with diffuse RPE dystrophy and peripheral BSP. FAF imaging showing focal areas of reduced autofluorescence in mid-periphery and at the macular area. OCT scan showing RPE dystrophy sparing the fovea. **g** Patient P10-GR: Fundus photograph showing diffuse RPE dystrophy with BSP. FAF shows focal areas of absent autofluorescence in mid-periphery and in the macular area. Macular SD-OCT scan shows RPE atrophy.

**Fig. 3:**
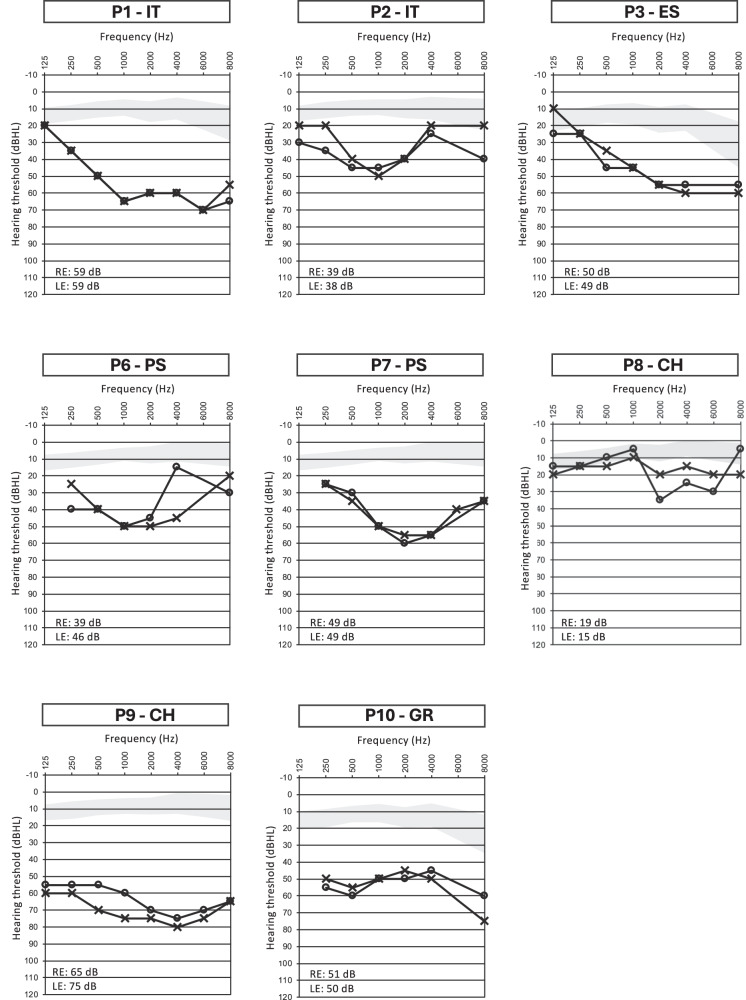
Pure-tone audiometry assessments in the *AGBL5* patients. Air conduction hearing thresholds of patients with biallelic variants in *AGBL5* in the frequency range 125 Hz–8000 Hz. Air conduction pure-tone average values for the right (RE) and left ear (LE) are reported at the bottom part of each audiogram. *AGBL5* patients showed a moderate hearing loss. The gray-shaded areas represent the hearing threshold average (± 1 SD) in sex- and age (decade)-matched normal individuals, as reported in a large retrospective study by Wasano et al. [34]. In each proband, air conduction and bone conduction thresholds (not depicted) overlapped. Symbols: O, air conduction in right ear; X, air conduction in left ear, dBHL, decibels hearing level.

The patient P1-IT is a 55 year-old female sporadic case referred for genetic testing with a clinical diagnosis of Usher syndrome (Table 1). She is the second of two sisters born to reportedly non-consanguineous parents from a small local community. Her medical history is insignificant with no cases of hearing loss and/or RP in the family (Fig. 1a). The patient presented night blindness since the age of 14 years, and received a diagnosis of RP at the age of 19 years. At the last ophthalmological examination (55 years), she had a hand motion visual acuity in both eyes, an RP fundus with bone-spicule pigmentation (BSP) in mid-periphery, and macular atrophy (Fig. 2a). FAF imaging revealed reduced autofluorescence at the posterior pole, with focal areas of absent autofluorescence surrounding the optic disc and in the macular area (Fig. 2a). SD-OCT imaging revealed a diffuse retinal pigment epithelium (RPE) dystrophy with epiretinal membranes but without cystoid macular edemas (CME) (Fig. 2a). Scotopic and photopic ERG responses were unrecordable in both eyes (Table 2), and she had bilateral pseudophakia. She also suffers from post-verbal hearing loss with a reported onset at the age of 18 years and she is currently using hearing aids (auditory prostheses). The most recent audiological examination confirmed the diagnosis of moderate, bilateral and symmetric sensorineural hearing loss (SNHL) with a Pure Tone Average (PTA) of 59 dB in both sides and a sloping audiogram at intermediate-high frequencies (500 Hz–8000 Hz) (Fig. 3). The hearing loss appeared non-progressive over the years. Clinical exome sequencing (Custom Constitutional Panels, Agilent; 5228 genes) revealed a homozygous missense variant at c.323C>G, p.(Pro108Arg) in the *AGBL5* gene (NM_021831.6), predicted to replace a highly conserved proline residue with arginine (Table 1, Fig. 1a). The variant is not present in reference population databases (e.g. gnomAD) but had already been reported as causative in patients with isolated RP [7] (Fig. 1i). The variant is classified as Likely pathogenic according to the American College of Medical Genetics (ACMG) criteria [26] and was interpreted as such in ClinVar (VCV001184585.2).

### Index case P2-IT (family F2)

The patient P2-IT is a 42-year-old male with a diagnosis of Usher syndrome (Table 1). He is the fifth of six offsprings born to consanguineous parents (Fig. 1b). His eldest brother (II.1) presents RP and profound prelingual hearing loss, which was left untreated compromising his speaking skills. The proband (II.5) reported onset of visual problems at 14 years of age and received a diagnosis of RP with macular involvement. In the most recent ophthalmological examination at the age of 42 years, he had a BCVA of 0.2 decimal (D) with a tubular 5° central visual field in the right eye, and hand motion perception in the left eye (Table 2). At fundus examination, he presented a pale optic disc, BSP in the mid-periphery, and diffuse RPE dystrophy (Fig. 2b). FAF imaging showed reduced autofluorescence at the posterior pole with focal areas of absent autofluorescence surrounding the optic disc (Fig. 2b), and SD-OCT revealed reduced central retina thickness and RPE atrophy at the macular area (Fig. 2b). He had bilateral pseudophakia and his ERG responses were unrecordable (Table 2). He also presents mild-to-moderate post-verbal hearing loss, bilateral and symmetric, with a PTA of around 39 dB in either side (Fig. 3). Quartet WES analysis with the samples of his unaffected parents (I.1, I.2) and affected brother (II.1) revealed a homozygous 2-bp deletion (c.184_185del) in *AGBL5*, predicted to cause a premature stop codon in amino acid position 63 (p.(Ala63Ter)) in the two affected sibs (Table 1, Fig. 1b). The variant was novel and was absent from population frequency databases (gnomAD, ExAc, 1,000 genomes), the Leiden Open Variation Database (LOVD) and ClinVar, and was classified as Likely Pathogenic according to the ACMG criteria (PVS1, PM2). Interestingly, his brother (II.1), besides being homozygous for the *AGBL5* variant, also harbored a homozygous, likely pathogenic, nonsense variant (NM_016239.4:c.7121C>G, p.(Ser2374Ter)) in *MYO15A*, a gene associated with profound, congenital, nonsyndromic deafness (OMIM * 602666). The proband was heterozygous for the *MYO15A* variant, as were his unaffected parents (Fig. 1b). Segregation analysis was extended to an unaffected sister (II.2) who was heterozygous for the *AGBL5* and *MYO15A* variants. Unfortunately, his brother was not willing to collaborate on the clinical part of the study, which would have allowed us to better define his apparently blended phenotype.

### Index case P3-ES (family F3)

F3 is a consanguineous family (parents are second cousins) of Spanish origin with three affected and two unaffected siblings (Fig. 1c). The index case (P3-ES; II.3) is a 58 years-old female with a typical RP and a late-onset, moderate, non-progressive hearing loss at high frequencies (Fig. 3). She had a BCVA of 0.3 D with a 10° central visual field in either eye, and presented a typical RP fundus with a pale optic disc, vessel attenuation, and pigment deposits in the periphery (with alterations of the RPE at the macular area). She had bilateral pseudophakia and did not present CME (Table 2). Her affected brothers were not collaborative for clinical examination in this study, but the proband referred that her elder brother (II.1) uses hearing aids and her younger brother (II.5) has a mild hearing impairment, both presenting also RP. Her unaffected siblings (II.2, II.4) do not present RP or hearing impairment. The three affected members were initially referred for genetic testing in 2015 with a clinical diagnosis of isolated RP. One of them (II.3, P3-ES) was analysed using a custom panel of 117 genes associated with IRDs, but no pathogenic variants were identified [27]. Subsequently, WES performed in two affected siblings (II.3, II.5) revealed compound heterozygous pathogenic variants in *AGBL5* (Table 1) which segregated with the disease in the family (Fig. 1c). The variant c.1565del, p.(Gly522AspfsTer4) was absent from population databases and was classified as Likely Pathogenic considering the ACMG criteria PVS1 and PM2. The missense variant c.908G>A, p.(Arg303His) had an allele frequency (AF) of 0.0000233 in gnomAD (AF = 0.0000269 in European non-Finnish) and was found at homozygous state in two unrelated patients of this study (P8-CH, P9-CH). The variant had a MutScore of 0.943 and was classified as Likely Pathogenic based on PP3_strong, PM2, PM3.

### Index case P6-PS (family F4)

P6-PS is a 26-year-old male patient and was the only affected individual in a consanguineous family of Palestinian origin (Fig. 1d). He presented the clinical features of RP, with vessel attenuation, RPE dystrophy without BSP, and CME in both eyes (Fig. 2d). He had a visual acuity of 0.5 D in both eyes, posterior subcapsular cataract and unrecordable ERG responses (Table 2). He also complained of mild-to-moderate hearing loss at low to mid frequencies (Fig. 3). The patient was homozygous for the nonsense variant c.170G>A, p.(Trp57Ter) in *AGBL5* (Fig. 1d, Table 1), which segregated with the disease in the family. The variant was novel, classified as Likely Pathogenic based on the ACMG criteria (PVS1, PM2), and was absent from population databases, but identified in another unrelated patient of Palestinian origin (P7-PS) in this study cohort.

### Index case P7-PS (family F5)

F5 is a consanguineous family of Palestinian origin with a 28 years-old affected male (P7-PS). The patient had a typical RP fundus with RPE dystrophy and BSP (Fig. 2e). He had a visual acuity of 0.16 D in both eyes, posterior subcapsular cataract and unrecordable ERG responses (Table 2). SD-OCT revealed a paracentral photoreceptor layer atrophy but no CME (Fig. 2e). He also complained of bilateral, symmetric SNHL of a moderate degree with a PTA of 48 dB in both ears (Fig. 3). The patient was homozygous for the c.170G>A, p.(Trp57Ter) variant (Fig. 1e, Table 1), which properly segregated with the disease in the family.

### Index case P8-CH (family F6)

This proband started experiencing a decrease in visual acuity since her teens, followed by night blindness, and was referred for an ophthalmological assessment at the age of 16 years. She was from a non-consanguineous family, however both parents came from the same village (Fig. 1f). At the first visit, her BCVA was 0.3 D in both eyes and the scotopic ERG responses were severely reduced, while the 30-Hz Flicker had reduced amplitude. At her last visit, aged 38 years, her BCVA was 0.1 D in both eyes, and the GVF was reduced to 30° bilaterally. FAF imaging showed several areas of patchy hypoautofluroescence at the posterior pole, which increased over time. The OCT scans at the last visit showed reduced retinal thickness with a severely altered layer of RPE and an absent ellipsoid layer at the posterior pole (Fig. 2f, Table 2). She never complained of hearing problems, however, the audiogram perfomed when she was 36 years old showed a reduced hearing threshold at higher frequencies (between 2000 and 4000 Hz) to 35 dB in the right ear and to 20 dB in the left side (Fig. 3, Table 2). No other family member had vision problems, however her younger sister (II.3) had severe hearing impairment (Fig. 1f). The genetic testing of the proband revealed a homozygous missense variant c.908G>A, p.(Arg303His) in the *AGBL5* gene (Table 1, Fig. 1f). The sister who presented isolated severe hearing loss was heterozygous for the *AGBL5* variant but was found to be compound heterozygous for two variants (c.35del and c.2229T>C) in the *GJB2* gene, explaining her hearing impairment (Fig. 1f). The proband did not carry any of the *GJB2* variants.

### Index case P9-CH (family F7)

P9-CH is a 39-year-old male patient diagnosed with RP and congenital hearing loss. He has an affected brother and an unaffected sister (Fig. 1f). The brother (II.1) was not available for the study, but the proband mentioned that his brother was less severely affected than him despite being 15 years older. At the time of the first referral to the clinic, the patient presented with a monocular situation after enucleation of the right eye due to painful phthisis bulbi, which he developed after multiple surgeries to treat retinal detachment when he was 18 and 20 years old. Fundus examination of the left eye revealed typical RP with waxy optic nerve head pallor, attenuated arteries, and peripheral BSP and retinal atrophy (Fig. 2f). No CME was observed at the most recent visit or previously described in his medical history. The patient had a visual field of <10° as assessed with a microperimetry device (Nidek MP3), which corresponds well to the margins of the retinal degeneration. He also presented subcapsular cataract and received cataract surgery. Following the surgery, his BCVA in the left eye increased from 0.3 D to 1.5 D. The patient reported hearing impairment since childhood (Fig. 3). Audiological testing indicated a bilateral sensorineural hearing loss with a flat audiogram. Molecular analysis revealed a homozygous variant in *AGBL5* (c.908G>A, p.(Arg303His)) (Table 1, Fig. 1f).

### Index case P10-GR (family F8)

P10-GR is a 63 year-old female with adulthood-onset RP. She is the third offspring of a non-consanguineous marriage with no family history of RP or hearing loss (Fig.1g). She reported night blindness since the age of 25 years with subsequent visual field constriction around the age of 35 and recent onset of photophobia. Her BCVA was 0.16 D in the right eye and 0.125 D in the left eye and she was pseudophakic. Fundoscopy revealed the presence of diffuse outer retinal atrophy with intraretinal pigment migration in the mid- and far-periphery along with foveal and peripapillary atrophy and vessel attenuation. FAF imaging showed areas of hypo-autofluorescence in the periphery, around the optic disc and in the fovea corresponding to the areas of retinal atrophy. SD-OCT showed retinal thinning with atrophy of outer retinal layers and foveal RPE loss (Fig. 2f, Table 2). The patient reported bilateral hearing impairment since the age of 58 years. Audiometry confirmed the presence of moderate SNHL with a flat audiogram and a PTA of 50 dB (Fig. 3).

Molecular analysis identified two compound heterozygous variants in *AGBL5*: c.883G>A, p.(Asp295Asn) and c.316A>G, p.(Met106Val), that were shown to be in trans, since the unaffected daughter of the patient only carries the c.883G>A, p.(Asp295Asn) (Fig. 1g, Table 1). The c.883G>A, p.(Asp295Asn) variant had an allele frequency (AF) of 0.00000272 in gnomAD and was reported as Likely Pathogenic in the ClinVar database (VCV000242932.5). The change was previously identified at homozygous state in three Turkish siblings with RP [5]. The c.316A>G, p.(Met106Val) was a novel variant that was absent from ClinVar and LOVD (AF of 0.00000223 in gnomAD), and was classified as VUS (variant of uncertain significance) according to the ACMG criteria (PM2, PM3, PP3). The variant had a MutScore of 0.682 and was predicted to replace a highly conserved methionine.

## Discussion

Syndromic IRDs constitute a range of disorders often characterized by the simultaneous presence of hearing loss and retinal degeneration, with Usher syndrome representing a predominant entity [28]. Usher patients are typically classified into three main clinical subtypes, namely Usher type I, II and III, based on the severity of hearing loss, age of onset, and vestibular involvement [28]. Recently, an Usher clinical type IV (OMIM #618144) has been defined, encompassing patients with late onset of symptoms (RP and progressive SNHL) and without vestibular impairment [29–31]. Nevertheless, some cases manifesting retinal disease and SNHL do not align with the classification criteria for the typical Usher subtypes and are referred to as ‘Usher-like’ patients. So far, eleven causative genes have been associated with the pathogenesis of Usher types I–IV, while additional genes, including *CEP250* and *CEP78*, are reported to cause atypical Usher forms or deafness-blindness syndromes [32]. This broader genetic landscape warrants further investigation for a comprehensive understanding of the mechanisms underlying atypical Usher phenotypes. In this study, we demonstrate that biallelic pathogenic variants in *AGBL5*, previously linked only to isolated RP, can induce a syndromic Usher-like phenotype, encompassing both RP and hearing loss. To our knowledge, only a very recent report described two sisters with RP and SNHL, carrying a homozygous missense variant in *AGBL5*, further supporting the herein proposed association of *AGBL5* mutations with syndromic RP [33].

There are over 60 pathogenic *AGBL5* variants reported in the Human Gene Mutation Database Professional (HGMD; https://www.hgmd.cf.ac.uk/ac/index.php). In our cohort, two variants are recurrent. The variant c.908G>A is present in the Spanish and the two Swiss families (at heterozygous state in P3-ES and at homozygous state in P8-CH and P9-CH), while the variant c.170G>A is present at homozygous state in the two Palestinian families. It is tempting to speculate that these variants either have a common ancestor or represent mutational hot-spots. However, the low number of cases with mutations in *AGBL5* makes it challenging to discriminate between these two possibilities. We studied this syndromic phenotype in eight unrelated families from diverse geographic backgrounds/origins. Affected individuals exhibited progressive retinal degeneration attributed to RP with onset in adolescence or early adulthood. Macular atrophy was observed in a significant subset of our patients (P1, P2, P7, P9), therefore placing *AGBL5* among the genes linked to RP with early macular involvement. Moreover, all patients presented cataract. Although lens abnormalities are a common feature in RP, further studies in large cohorts are necessary to assess whether *AGBL5*-associated subjects have an increased likelihood of developing such defects. Additionally, all patients presented symmetric, mild-to-moderate SNHL without noticeable fluctuations over time. The hearing thresholds of the *AGBL5* patients, at all frequencies tested, (with the exception of low frequencies 125 Hz-1000 Hz for P8-CH) were higher than the normal range observed in large age- and sex-matched cohorts without ear disease (Fig. 3) [34]. The reported onset of hearing loss varied, ranging from birth in one case to 58 years of age, and notably, lacked vestibular symptoms. The association of *AGBL5* variants with this deaf-blindness syndrome may have not been recognized earlier possibly due to the infrequent occurrence of variants in the *AGBL5* gene. Given the estimated carrier frequency of *AGBL5* variants in the European (non-Finnish) population, reported by Hanany et al., the expected number of individuals with *AGBL5*-associated disease is nearly 21 times lower compared to *MYO7A-*related conditions and roughly 175 times smaller than the anticipated cases linked to *USH2A* [35]. Additionally, the targeted analyses of Usher patients using restricted disease-associated panels may have hindered the earlier identification of such genotype-phenotype association. Furthermore, considering that the hearing impairment observed in *AGBL5* patients was both late-onset and relatively mild, it cannot be ruled out that previously reported *AGBL5* cases with isolated RP may have had a hearing deficit that went unnoticed during ophthalmological assessment, especially when individuals do not exhibit noticeable symptoms or if the symptoms are subtle, or may have developed hearing loss later in the disease course. It is therefore advisable to refer patients with pathogenic variants in *AGBL5* for audiological testing to detect any potential mild hearing deficits.

*AGBL5* encodes a cilia-enriched α-tubulin deglutamylase which specifically catalyses the removal of the branching point of glutamylated tubulin [15, 17]. Dysfunction of AGBL5 results in alterations to the complex glutamylation patterns of tubulin, impacting the binding capacity and functional interactions of microtubules [5, 7, 8, 36]. Proper glutamylation of tubulin is essential for maintaining the function of sensory cilia in the retina and inner ear. Consequently, mutations in *AGBL5* may lead to ciliopathy phenotypes with extra-retinal manifestations [5]. Previously, other studies have demonstrated that alteration of tubulin structures in the inner ear can also cause hearing loss [37]. Pathogenic variants in the *TBCE* gene are linked to a disruption of tubulin structures in the auditory nerve, as well as to a progressive loss of outer hair cells [38]. Similarly, variants in *TUBB4B* have been associated with autosomal dominant Leber Congenital Amaurosis and Early Onset Deafness (LCAEOD) [39, 40]. The multifaceted functions of microtubules, subject to tissue-specific post-translational modifications, underscore the need for proper expression and function of AGBL5 to maintain specific tubulin structures in ciliated cells of the retina and inner ear. The importance of cilia in the broader context of genetic disorders affecting vision and hearing is evident by the involvement of numerous Usher-associated genes (i.e. *MYO7A*, *USH2A*, *USH1C*, *USH1G*, *PCDH15*, *CDH23*), including those proposed for atypical forms (i.e. *CEP250*) or deafness-blindness syndromes (e.g. *CEP78*), in ciliary function. For instance, *CEP78* encodes a centrosomal protein involved in cilia formation and in the regulation of ciliary length, and is responsible for a cone-rod dystrophy with early onset SNHL [41, 42]. Similarly, CEP250 is localized in cilia and its dysfunction has been associated with a moderate form of RP with progressive SNHL or with a cone-rod dystrophy combined with deafness [43, 44].

In conclusion, the analysis of the sensory phenotype of patients with biallelic variants in *AGBL5* has expanded our understanding of the gene’s implication in the context of inherited retinal disorders. We identified seven different *AGBL5* variants in our cohort, four of which are novel. We did not observe any correlation between the variant type or location and the manifestation of syndromic or isolated RP (Fig. 1i). The complex relationship between genotypic variations in *AGBL5* and the phenotypic expression of hearing impairment emphasizes the need for further investigations in larger cohorts to elucidate the underlying mechanisms. Similar studies have already been undertaken for *USH2A* cohorts without establishing a clear correlation between genotypes and isolated or syndromic IRD forms [45]. The identification of *AGBL5* as a causative gene of Usher-like phenotypes advances our understanding of the intricate manifestations of Usher syndrome and highlights the pathogenic role of ‘tubulin code’ perturbations in sensory ciliopathies.

## Supplementary information

Supplementary Methods

## Data Availability

The datasets generated and/or analysed during the current study are available from the corresponding author on reasonable request.

## References

[1] Hartong DT, Berson EL, Dryja TP. Retinitis pigmentosa. Lancet. 2006;368:1795–809. 17113430 10.1016/S0140-6736(06)69740-7

[2] Chang S, Vaccarella L, Olatunji S, Cebulla C, Christoforidis J. Diagnostic challenges in retinitis pigmentosa: genotypic multiplicity and phenotypic variability. Curr Genom. 2011;12:267–75. 10.2174/138920211795860116 PMC3131734 22131872

[3] Hamel C. Retinitis pigmentosa. Orphanet J Rare Dis. 2006;1:40. 17032466 10.1186/1750-1172-1-40 PMC1621055

[4] Tatour Y, Ben-Yosef T. Syndromic inherited retinal diseases: genetic, clinical and diagnostic aspects. Diagnostics. 2020;10(Oct):779. 33023209 10.3390/diagnostics10100779 PMC7600643

[5] Kastner S, Thiemann IJ, Dekomien G, Petrasch-Parwez E, Schreiber S, Akkad DA, et al. Exome sequencing reveals agbl5 as novel candidate gene and additional variants for retinitis pigmentosa in five turkish families. Invest Ophthalmol Vis Sci. 2015;56:8045–53. 26720455 10.1167/iovs.15-17473

[6] Patel N, Aldahmesh MA, Alkuraya H, Anazi S, Alsharif H, Khan AO, et al. Expanding the clinical, allelic, and locus heterogeneity of retinal dystrophies. Genet Med. 2016;18:554–62. 26355662 10.1038/gim.2015.127

[7] Astuti GDN, Arno G, Hull S, Pierrache L, Venselaar H, Carss K, et al. Mutations in AGBL5, encoding α-tubulin deglutamylase, are associated with autosomal recessive retinitis pigmentosa. Invest Ophthalmol Vis Sci. 2016;57:6180–7. 27842159 10.1167/iovs.16-20148

[8] Branham K, Matsui H, Biswas P, Guru AA, Hicks M, Suk JJ, et al. Establishing the involvement of the novel gene AGBL5 in retinitis pigmentosa by whole genome sequencing. Physiol Genom. 2016;48:922–7. 10.1152/physiolgenomics.00101.2016 PMC5206392 27764769

[9] Abu Diab A, AlTalbishi A, Rosin B, Kanaan M, Kamal L, Swaroop A, et al. The combination of whole-exome sequencing and clinical analysis allows better diagnosis of rare syndromic retinal dystrophies. Acta Ophthalmol. 2019;97(Sep):e877–86. 30925032 10.1111/aos.14095 PMC11377105

[10] Khan AO. Phenotype-guided genetic testing of pediatric inherited retinal disease in the United Arab Emirates. Retina. 2020;40:1829–37. 31725702 10.1097/IAE.0000000000002675

[11] Sun Y, Li W, Li JK, Wang ZS, Bai JY, Xu L, et al. Genetic and clinical findings of panel-based targeted exome sequencing in a northeast Chinese cohort with retinitis pigmentosa. Mol Genet Genom Med. 2020;8:e1184. 10.1002/mgg3.1184 PMC7196472 32100970

[12] Méjécase C, Kozak I, Moosajee M. The genetic landscape of inherited eye disorders in 74 consecutive families from the United Arab Emirates. Am J Med Genet C Semin Med Genet. 2020;184:762–72. 32783370 10.1002/ajmg.c.31824 PMC8432150

[13] Turro E, Astle WJ, Megy K, Gräf S, Greene D, Shamardina O, et al. Whole-genome sequencing of patients with rare diseases in a national health system. Nature. 2020;583(Jul):96–102. 32581362 10.1038/s41586-020-2434-2 PMC7610553

[14] Paredes DI, Bello NR, Capasso JE, Procopio R, Levin AV. Mutations in AGBL5 associated with Retinitis pigmentosa. Ophthalmic Genet. 2024;45:275–80. 10.1080/13816810.2023.2291687 38078364

[15] Rogowski K, van Dijk J, Magiera MM, Bosc C, Deloulme JC, Bosson A, et al. A family of protein-deglutamylating enzymes associated with neurodegeneration. Cell. 2010;143(Nov):564–78. 21074048 10.1016/j.cell.2010.10.014

[16] Berezniuk I, Lyons PJ, Sironi JJ, Xiao H, Setou M, Angeletti RH, et al. Cytosolic carboxypeptidase 5 removes α- and γ-linked glutamates from tubulin. J Biol Chem. 2013;288:30445–53. 24022482 10.1074/jbc.M113.497917 PMC3798508

[17] Kimura Y, Kurabe N, Ikegami K, Tsutsumi K, Konishi Y, Kaplan OI, et al. Identification of tubulin deglutamylase among Caenorhabditis elegans and mammalian cytosolic carboxypeptidases (CCPs). J Biol Chem. 2010;285:22936–41. 20519502 10.1074/jbc.C110.128280 PMC2906286

[18] Yang WT, Hong SR, He K, Ling K, Shaiv K, Hu J, et al. The emerging roles of axonemal glutamylation in regulation of cilia architecture and functions. Front Cell Dev Biol. 2021;9:622302. 33748109 10.3389/fcell.2021.622302 PMC7970040

[19] Pathak N, Austin-Tse CA, Liu Y, Vasilyev A, Drummond IA. Cytoplasmic carboxypeptidase 5 regulates tubulin glutamylation and zebrafish cilia formation and function. Mol Biol Cell. 2014;25:1836–44. 24743595 10.1091/mbc.E13-01-0033 PMC4055263

[20] Lyons PJ, Sapio MR, Fricker LD. Zebrafish cytosolic carboxypeptidases 1 and 5 are essential for embryonic development. J Biol Chem. 2013;288:30454–62. 24022483 10.1074/jbc.M113.497933 PMC3798509

[21] Wu P, Zuo X, Deng H, Liu X, Liu L, Ji A. Roles of long noncoding RNAs in brain development, functional diversification and neurodegenerative diseases. Br Res Bull. 2013;97:69–80. 10.1016/j.brainresbull.2013.06.001 23756188

[22] Giordano T, Gadadhar S, Bodakuntla S, Straub J, Leboucher S, Martinez G, et al. Loss of the deglutamylase CCP5 perturbs multiple steps of spermatogenesis and leads to male infertility. J Cell Sci. 2019;132:jcs226951. 30635446 10.1242/jcs.226951

[23] Xia P, Ye B, Wang S, Zhu X, Du Y, Xiong Z, et al. Glutamylation of the DNA sensor cGAS regulates its binding and synthase activity in antiviral immunity. Nat Immunol. 2016;17:369–78. 26829768 10.1038/ni.3356

[24] He K, Ma X, Xu T, Li Y, Hodge A, Zhang Q, et al. Axoneme polyglutamylation regulated by Joubert syndrome protein ARL13B controls ciliary targeting of signaling molecules. Nat Commun. 2018;9:3310. 30120249 10.1038/s41467-018-05867-1 PMC6098020

[25] Marmor MF, Fulton AB, Holder GE, Miyake Y, Brigell M, Bach M, et al. ISCEV Standard for full-field clinical electroretinography (2008 update). Doc Ophthalmol. 2009;118:69–77. 19030905 10.1007/s10633-008-9155-4

[26] Richards S, Aziz N, Bale S, Bick D, Das S. ACMG Standards and Guidelines Standards and guidelines for the interpretation of sequence variants: a joint consensus recommendation of the American College of Medical Genetics and Genomics and the Association for Molecular Pathology. Genet Med. 2015:1–20. 10.1038/gim.2015.30 PMC4544753 25741868

[27] Rodríguez-Muñoz A, Aller E, Jaijo T, González-García E, Cabrera-Peset A, Gallego-Pinazo R, et al. Expanding the clinical and molecular heterogeneity of nonsyndromic inherited retinal dystrophies. J Mol Diagn. 2020;22:532–43. 32036094 10.1016/j.jmoldx.2020.01.003

[28] Fuster-García C, García-Bohórquez B, Rodríguez-Muñoz A, Aller E, Jaijo T, Millán JM, et al. Usher syndrome: genetics of a human ciliopathy. IJMS. 2021;22:6723. 34201633 10.3390/ijms22136723 PMC8268283

[29] Abad-Morales V, Navarro R, Burés-Jelstrup A, Pomares E. Identification of a novel homozygous ARSG mutation as the second cause of Usher syndrome type 4. Am J Ophthalmol Case Rep. 2020;19:100736. 32455177 10.1016/j.ajoc.2020.100736 PMC7235610

[30] Peter VG, Quinodoz M, Sadio S, Held S, Rodrigues M, Soares M, et al. New clinical and molecular evidence linking mutations in ARSG to Usher syndrome type IV. Hum Mutat. 2021;42:261–71. 33300174 10.1002/humu.24150

[31] Velde HM, Reurink J, Held S, Li CHZ, Yzer S, Oostrik J, et al. Usher syndrome type IV: clinically and molecularly confirmed by novel ARSG variants. Hum Genet. 2022;141:1723–38. 35226187 10.1007/s00439-022-02441-0 PMC9556359

[32] Igelman AD, Ku C, da Palma MM, Georgiou M, Schiff ER, Lam BL, et al. Expanding the clinical phenotype in patients with disease causing variants associated with atypical Usher syndrome. Ophthalmic Genet. 2021;42:664–73. 34223797 10.1080/13816810.2021.1946704 PMC9233901

[33] Holanda IP, Rim PHH, Rare Genomes Project Consortium null, Guaragna MS, Gil-da-Silva-Lopes VL, Steiner CE. Syndromic Retinitis Pigmentosa: A 15-Patient Study. Genes. 2024;15:516. 38674450 10.3390/genes15040516 PMC11050127

[34] Wasano K, Kaga K, Ogawa K. Patterns of hearing changes in women and men from denarians to nonagenarians. Lancet Reg Health West Pac. 2021;9:100131. 34327440 10.1016/j.lanwpc.2021.100131 PMC8315603

[35] Hanany M, Rivolta C, Sharon D. Worldwide carrier frequency and genetic prevalence of autosomal recessive inherited retinal diseases. Proc Natl Acad Sci USA. 2020;117:2710–6. 31964843 10.1073/pnas.1913179117 PMC7007541

[36] McKenna ED, Sarbanes SL, Cummings SW, Roll-Mecak A. The tubulin code, from molecules to health and disease. Annu Rev Cell Dev Biol. 2023;39:331–61. 37843925 10.1146/annurev-cellbio-030123-032748

[37] Deretic J, Odabasi E, Firat-Karalar EN. The multifaceted roles of microtubule-associated proteins in the primary cilium and ciliopathies. J Cell Sci. 2023;136:jcs261148. 38095645 10.1242/jcs.261148

[38] Rak K, Frenz S, Radeloff A, Groh J, Jablonka S, Martini R, et al. Mutation of the TBCE gene causes disturbance of microtubules in the auditory nerve and cochlear outer hair cell degeneration accompanied by progressive hearing loss in the PMN/PMN mouse. Exp Neurol. 2013;250:333–40. 24120439 10.1016/j.expneurol.2013.10.007

[39] Luscan R, Mechaussier S, Paul A, Tian G, Gérard X, Defoort-Dellhemmes S, et al. Mutations in TUBB4B cause a distinctive sensorineural disease. Am J Hum Genet. 2017;101:1006–12. 29198720 10.1016/j.ajhg.2017.10.010 PMC5812887

[40] Maasz A, Hadzsiev K, Ripszam R, Zsigmond A, Maka E, Knezy K, et al. TUBB4B gene mutation in Leber phenotype of congenital amaurosis syndrome associated with early-onset deafness. Eur J Med Genet. 2022;65:104471. 35240325 10.1016/j.ejmg.2022.104471

[41] Namburi P, Ratnapriya R, Khateb S, Lazar CH, Kinarty Y, Obolensky A, et al. Bi-allelic truncating mutations in CEP78, Encoding Centrosomal Protein 78, cause cone-rod degeneration with sensorineural hearing loss. Am J Hum Genet. 2016;99:1222–3. 27588452 10.1016/j.ajhg.2016.07.010 PMC5011076

[42] Nikopoulos K, Farinelli P, Giangreco B, Tsika C, Royer-Bertrand B, Mbefo MK, et al. Mutations in CEP78 cause cone-rod dystrophy and hearing loss associated with primary-cilia defects. Am J Hum Genet. 2016;99:770–6. 27588451 10.1016/j.ajhg.2016.07.009 PMC5011074

[43] Fuster-García C, García-García G, Jaijo T, Fornés N, Ayuso C, Fernández-Burriel M, et al. High-throughput sequencing for the molecular diagnosis of Usher syndrome reveals 42 novel mutations and consolidates CEP250 as Usher-like disease causative. Sci Rep. 2018;8:17113. 30459346 10.1038/s41598-018-35085-0 PMC6244211

[44] Khateb S, Zelinger L, Mizrahi-Meissonnier L, Ayuso C, Koenekoop RK, Laxer U, et al. A homozygous nonsense CEP250 mutation combined with a heterozygous nonsense C2orf71 mutation is associated with atypical Usher syndrome. J Med Genet. 2014;51:460–9. 24780881 10.1136/jmedgenet-2014-102287

[45] Lenassi E, Vincent A, Li Z, Saihan Z, Coffey AJ, Steele-Stallard HB, et al. A detailed clinical and molecular survey of subjects with nonsyndromic USH2A retinopathy reveals an allelic hierarchy of disease-causing variants. Eur J Hum Genet. 2015;23:1318–27. 25649381 10.1038/ejhg.2014.283 PMC4592079

