# Supplementary material for: Variants in the AGBL5 gene are responsible for autosomal recessive Retinitis pigmentosa with hearing loss
Source: Eur J Hum Genet. 2024 Dec 13;33(6):727–37. doi: 10.1038/s41431-024-01768-8 (PMC12185745; doi:10.1038/s41431-024-01768-8)
Supplement: Supplementary file 1 — Supplementary Methods [file 41431_2024_1768_MOESM1_ESM.docx]

**SUPPLEMENTARY MATERIAL**

**Variants in *AGBL5* gene are responsible for autosomal recessive Retinitis pigmentosa with hearing loss**

Marianthi Karali^1,2*^, Gema García-García^3,4,5*^, Karolina Kaminska^6^, Alaa AlTalbishi^7^, Francesca Cancellieri^6^, Francesco Testa^2^, Maria Rosaria Barillari^8^, Evangelia S. Panagiotou^9,10^, George Psillas^11^, Veronika Vaclavik^12^, Viet H. Tran^12,13^, Lucas Janeschitz-Kriegl^14^, Hendrik PN Scholl^14^, Manar Salameh^7^, Pilar Barberán-Martínez^3,5^, Ana Rodriguez-Muñoz^15^, Miguel Armengot^16^, Margherita Scarpato^1^, Roberta Zeuli^1^, Mathieu Quinodoz^6,17^, Francesca Simonelli^2^, Carlo Rivolta^6,17^, Sandro Banfi^1,18†^, José M. Millán^3,4,5,9†^

^1^ Medical Genetics, Department of Precision Medicine, University of Campania 'Luigi Vanvitelli', 80138, Naples, Italy

^2^ Eye Clinic, Multidisciplinary Department of Medical, Surgical and Dental Sciences, University of Campania 'Luigi Vanvitelli', 80131, Naples, Italy

^3^ Molecular, Cellular, and Genomic Biomedicine Group, IIS-La Fe, Valencia, Spain

^4^ Center for Rare Diseases (CIBERER), Madrid, Spain

^5^ Joint Unit CIPF-IIS La Fe Molecular, Cellular, and Genomic Biomedicine, Valencia, Spain

^6^ Institute of Molecular and Clinical Ophthalmology Basel, 4031 Basel, Switzerland

^7^ St John of Jerusalem Eye Hospital, Jerusalem, Palestine

^8^Department of Mental and Physical Health and Preventive Medicine, University of Campania 'Luigi Vanvitelli', 80138, Naples, Italy

^9^ 1st Department of Ophthalmology, Aristotle University of Thessaloniki, AHEPA Hospital, Thessaloniki, Greece

^10^ Department of Ophthalmology, Ghent University Hospital, Ghent, Belgium

^11^ 1st Academic ENT Department, School of Medicine, Aristotle University of Thessaloniki, AHEPA Hospital, Thessaloniki, Greece)

^12^ Jules-Gonin Eye Hospital, Fondation Asile des Aveugles, University of Lausanne, 1004 Lausanne, Switzerland

^13^ Centre for Gene Therapy and Regenerative Medicine, King’s College London, London, UK

^14^ Department of Ophthalmology, University of Basel, Basel, Switzerland

^15^ University Dr Peset Hospital of Valencia, Spain

^16^ University and Polytechnic La Fe Hospital of Valencia, Spain

^17^ Department of Genetics and Genome Biology, University of Leicester, Leicester, UK

^18^ Telethon Institute of Genetics and Medicine, 80078 Pozzuoli, Italy

**Supplementary Methods**

**Exome sequencing and variant prioritisation approach**

The sequencing protocols used for each index case are specified below.

**P1-IT, P2-IT:** Genomic DNA was extracted from peripheral blood using the DNeasy Blood & Tissue Kit (QIAGEN, Venlo, Netherlands) according to the manufacturer’s instructions. Clinical exome (for P1-IT) and whole-exome sequencing (WES) libraries (for P2-IT) were prepared using the ClearSeq Inherited Disease Panel and the SureSelect Human Ali Exon v7 (Agilent, Santa Clara, CA, USA), respectively. Libraries were run on a NextSeq500 sequencing platform (Illumina inc., San Diego, CA, USA). Sequencing data were analysed using previously described pipeline(1).

**P3-ES:** Genomic DNA from the patients and relatives was obtained and purified using the automated DNA extractor QIAsymphony (QIAGEN, Venlo, Netherlands). The concentration of the resulting DNA samples was determined with Nanodrop and Qubit fluorometer (Thermo Fisher Scientific, Waltham, MA, USA). WES librariers were prepared using the Select Human All Exome V6 kit (Agilent, Santa Clara, CA, USA). Raw data were analysed using Sophia DMM software (Sophia Genetics, Lausanne, Switzerland).

**P6-PS, P7-PS:** Genomic DNA was extracted from peripheral blood using the Maxwell Promega automated extractor according to the manufacturer’s instructions. DNA sample concentration was measured by the Nanodrop. WES was done by the 3billion company (Seoul, Korea) on an Illumina NovaSeq6000 (Illumina inc., San Diego, CA, USA). Selected variants were validated by Sanger sequencing.

**P8-CH, P9-CH, P10-GR:** Genomic DNA was obtained from peripheral whole-blood for the Swiss patients, and from saliva samples for the Greek patient. WES was performed at CeGaT GmbH (Tübingen, Germany). There, sequencing libraries were generated using the Twist Human Core Exome Plus kit (Twist Bioscience, South San Francisco, CA, USA), following manufacturer's protocols. Libraries underwent paired-end sequencing on a Novaseq 6000 platform (Illumina), resulting in sequences of 100 bases. Processing of the data was performed as previously described(2).

The possible pathogenic role of the identified variants was evaluated considering:

(a) Segregation of the identified variants with the disease in the family.

(b) Presence and/or frequency of the variant in available national and international genomic databases (e.g. dbSNP v137, ExaC (http://exac.broadinstitute.org), and gnomAD (http://gnomad.broadinstitute.org), 1,000 genomes project, the Human Gene Mutation Database (HGMD, http://www.hgmd.cf.ac.uk), Clinvar (http://www.ncbi.nlm.nih.gov/clinvar/), the Leiden Open Variation Database (LOVD, http://www.lovd.nl/LOVD) and in-house Exome Servers.

(c) *In silico* predictions of the potential effect of the new variant on protein. For missense variants, positional scores, the degree of cross-species conservation of the affected residue(3) as well as the effect of the amino acid change on the protein structure was assessed through different bioinformatics tools (MutScore, SIFT, Mutation Taster, Polyphen, etc. (4–8)).

**References**

1. Musacchia F, Ciolfi A, Mutarelli M, Bruselles A, Castello R, Pinelli M, et al. VarGenius executes cohort-level DNA-seq variant calling and annotation and allows to manage the resulting data through a PostgreSQL database. BMC Bioinformatics. 2018 Dec 12;19(1):477.

2. Peter VG, Kaminska K, Santos C, Quinodoz M, Cancellieri F, Cisarova K, et al. The first genetic landscape of inherited retinal dystrophies in Portuguese patients identifies recurrent homozygous mutations as a frequent cause of pathogenesis. PNAS Nexus. 2023 Mar;2(3):pgad043.

3. Goode DL, Cooper GM, Schmutz J, Dickson M, Gonzales E, Tsai M, et al. Evolutionary constraint facilitates interpretation of genetic variation in resequenced human genomes. Genome Res. 2010 Mar;20(3):301–10.

4. Adzhubei I, Jordan DM, Sunyaev SR. Predicting functional effect of human missense mutations using PolyPhen-2. Curr Protoc Hum Genet. 2013 Jan;Chapter 7:Unit7.20.

5. Kumar P, Henikoff S, Ng PC. Predicting the effects of coding non-synonymous variants on protein function using the SIFT algorithm. Nat Protoc. 2009;4(7):1073–81.

6. Liu X, Jian X, Boerwinkle E. dbNSFP: a lightweight database of human nonsynonymous SNPs and their functional predictions. Hum Mutat. 2011 Aug;32(8):894–9.

7. Schwarz JM, Rödelsperger C, Schuelke M, Seelow D. MutationTaster evaluates disease-causing potential of sequence alterations. Nat Methods. 2010 Aug;7(8):575–6.

8. Quinodoz M, Peter VG, Cisarova K, Royer-Bertrand B, Stenson PD, Cooper DN, et al. Analysis of missense variants in the human genome reveals widespread gene-specific clustering and improves prediction of pathogenicity. Am J Hum Genet. 2022 Mar 3;109(3):457–70.
